# Enucleation Is a Feasible Procedure for Well-Differentiated pNEN—A Matched Pair Analysis

**DOI:** 10.3390/cancers14102570

**Published:** 2022-05-23

**Authors:** Anna Nießen, Fabiola A. Bechtiger, Ulf Hinz, Magdalena Lewosinska, Franck Billmann, Thilo Hackert, Markus W. Büchler, Simon Schimmack

**Affiliations:** Department of General, Visceral and Transplantation Surgery, Heidelberg University Hospital, Im Neuenheimer Feld 420, 69120 Heidelberg, Germany; anna.niessen@med.uni-heidelberg.de (A.N.); fabiola.bechtiger@med.uni-heidelberg.de (F.A.B.); ulf.hinz@med.uni-heidelberg.de (U.H.); magdalena.lewosinska@med.uni-heidelberg.de (M.L.); franck.billmann@med.uni-heidelberg.de (F.B.); thilo.hackert@med.uni-heidelberg.de (T.H.); markus.buechler@med.uni-heidelberg.de (M.W.B.)

**Keywords:** pNEN, enucleation, formal resection, neuroendocrine tumors, surgery

## Abstract

**Simple Summary:**

Neuroendocrine neoplasms of the pancreas (pNEN) are rare malignancies with an increasing incidence rate. Optimal treatment strategies for non-functional and well-differentiated tumors in particular are still controversially discussed. Enucleation and formal oncological resection of pNEN are well-established operative treatment strategies. However, the risk of metastases requiring radical formal resection has to be weighed against the risk of overtreatment and potentially higher postoperative complication rates, especially in cases of well-differentiated pNEN, which carry a lower risk for the development of metastases in the future. Thus, this study compared enucleation and formal resection for well-differentiated non-functional pNEN. Postoperative complication rates and overall as well as disease-free survival were similar in the cohorts studied. Postoperative diabetes was developed significantly less often after enucleation. Thus, enucleation is a safe surgical procedure with good long-term outcomes for a selected group of patients.

**Abstract:**

The extent of surgical resection in the treatment of pancreatic neuroendocrine neoplasms (pNEN) is still controversial. This study aimed to evaluate the outcomes of enucleation for well-differentiated non-functional (nf) pNEN. Patients undergoing enucleation (2001–2020) were analyzed. Clinicopathological parameters, perioperative outcomes and survival were assessed. The analysis was performed as a nested case-control study and matched-pair analysis with formal resection. Sixty-one patients undergoing enucleation were identified. Compared to patients undergoing formal resection, enucleation was associated with a significantly shorter median length of operative time (128 (IQR 95–170) versus 263 (172–337) minutes, *p* < 0.0001) and a significantly lower rate of postoperative diabetes (2% versus 21%, *p* = 0.0020). There was no significant difference in postoperative pancreatic fistula rate (18% versus 16% type B/C, *p* = 1.0), Clavien−Dindo ≥ III complications (20% versus 26%, *p* = 0.5189), readmission rate (12% versus 15%, *p* = 0.6022) or length of hospital stay (8 (7–11) versus 10 (8–17) days, *p* = 0.0652). There was no 30-day mortality after enucleation compared to 1.6% (*n* = 1) after formal resection. 10-year overall survival (OS) and disease-free survival (DFS) was similar between the two groups (OS: 89% versus 77%, *p* = 0.2756; DFS: 98% versus 91%, *p* = 0.0873). Enucleation presents a safe surgical approach for well-differentiated nf-pNEN with good long-term outcomes for selected patients.

## 1. Introduction

Pancreatic neuroendocrine neoplasms (pNEN) are a heterogenous entity that have been observed with increasing incidence over the past years [1,2,3]. Approximately 80% of neuroendocrine neoplasms of the pancreas are non-functional (nf-pNEN) [4]. While in most malignancies formal oncological resection including the primary plus the associated lymph nodes is considered the gold standard for curative therapy [5,6], optimal treatment strategies in NEN generally and pNEN specifically are still being debated.

For functional pNEN (f-pNEN), recommendations for the preferred surgical approaches are clear. For insulinoma, representing the most common type of f-pNEN, parenchyma-sparing operations, such as enucleation, are state of the art [7] as their risk for malignancy, local invasion and distant metastases is low [8]. In contrast, 60% of gastrinomas are associated with lymph node or distant metastases at the time of diagnosis and thus, formal resection is recommended for these f-pNEN [8,9,10,11,12,13]. Nf-pNEN, however, clinically present very heterogeneous behaviour and postresection survival is difficult to predict. Several risk factors, such as tumor size and grading, that influence survival and recurrence in these tumors have been identified [14,15,16,17,18,19,20]. The prognostic impact of lymph node metastases—which can be present in up to 60% of nf-pNEN at the time of diagnosis and also occur in small, well-differentiated nf-pNEN—on overall and disease-free survival is also still controversially reported and thus, a focus of intensive research [14,21,22,23,24,25]. 

Generally, the type and extent of resection and the decision to perform a systematic lymphadenectomy (LND) is chosen according to clinicomorphological features such as tumor size and margin and the presence of lymph node metastases. For nf-pNEN > 2 cm, complete and curatively intended resection is state of the art for nf-pNEN and has been shown to be significantly associated with better survival compared to surveillance [8,26,27]. For nf-pNEN ≤ 2 cm, active surveillance is also being discussed as an alternative to surgical resection [8,13,28]. Recent data, however, have shown an increased survival rate in such patients after surgical resection [29,30]. Furthermore, formal resection is generally not recommended and parenchyma-sparing resections, such as enucleation or segmental resection, present an alternative surgical approach, especially for small and well-differentiated nf-pNEN [8]. The benefit of the preservation of functionally active endocrine and exocrine pancreatic tissue must be weighed against the potential risk of increased postoperative complication rates, such as postoperative pancreatic fistula (POPF) [31,32,33,34,35]. Studies comparing enucleation and formal resection for small and well-differentiated pNEN with regards to oncological outcomes are scarce.

Therefore, the aim of this study was to compare the short and long-term outcomes of enucleation for non-functional well-differentiated G1 and G2 pNEN in comparison to formally resected patients. 

## 2. Methods

### 2.1. Patient Identification

This study was approved by the ethics committee of the Medical Faculty of the University of Heidelberg (No. S-708/2019) in accordance with the precepts established by the Helsinki Declaration. All patients undergoing enucleation for non-functional G1 or G2 differentiated pNEN between 1 October 2001 and 31 December 2020 were identified from the prospectively maintained pancreatic database at the Department of General, Visceral and Transplantation Surgery, University Hospital of Heidelberg, Germany. Inclusion criteria: pathological diagnosis of a pancreatic NEN. Exclusion criteria: functional pNEN or G3 differentiation. Consent for the retrospective data analyses and collection of follow-up information was obtained from all patients.

### 2.2. Clinical Data and Surgical Approach

Patient data were extracted from the hospital’s digital electronic patient information system and the prospectively maintained database. 

In order to evaluate patients for suitability of enucleation, cross-sectional imaging is performed preoperatively. Our gold standard, in accordance with guidelines, is multi-slice 3-phase Hydro-CT or alternatively, MRI/MRCP [36,37]. If the cross-sectional imaging is unambiguous, we do not insist on the performance of EUS as in the hands of experienced pancreatic radiologists and with the increasing availability of quantitative CT evaluation, preoperative discrimination of at least G1 and G2 differentiation versus G3 differentiation is becoming a realistic tool [38,39,40,41,42]. However, if the cross-sectional imaging is not clear, EUS and FNA is carried out. Exclusion criteria for enucleation are G3 differentiation, size > 3 cm, local invasion or complications such as thrombosis of the splenic vein, suspicion of lymph node or distant metastases on preoperative imaging.

Parameters collected in order to characterize the study cohort included the following: age, sex, tumor location, body mass index (BMI), Physical Status Classification System by the American Society of Anaesthesiologists (ASA) and indication for surgery. 

For the evaluation of the surgical procedure and the peri- and post-operative period, the following data were gathered: type and length of operation, perioperative morbidity and complications, 30-day/in-hospital mortality and length of hospital stay. POPF was classified according to the definition of the International Study Group of Pancreatic Surgery (ISGPS) [43,44]. General postoperative complications were graded according to Dindo et al. [45]. Pathology data obtained included resection margin status, tumor size, hormone expression status, lymph node status and tumor stage and grade (based on the Ki-67(MIB1) proliferation index), in accordance with the WHO and AJCC/UICC classifications of malignant tumors. In pNEN resected before 2017, the grading was re-classified post-hoc according to the current WHO definition and the TNM stage was revised post-hoc according to the 8th edition in order to ensure comparability [46,47,48].

### 2.3. Follow-Up 

Follow-up data were collected until 1st April 2022. Patients were followed up via information obtained from our outpatient care units, external oncological follow-up, telephone interview of patients, relatives or general practitioners and lastly, through information obtained from the residents’ registration offices. Follow-up parameters obtained were the current state of disease, recurrences or the need for reoperations or reinterventions, the development of postoperative diabetes (need for oral medication or insulin substitution) and in cases of death, the cause of death, amongst others. Patients were evaluated until their latest oncological surveillance examination or until death. 

### 2.4. Matched Pair Analysis

A nested case-control study with a matched pair design was performed in order to analyze perioperative and long-term outcomes of enucleation for well-differentiated pNEN. Patients undergoing enucleation were matched 1:1 with patients undergoing formal resection (distal pancreatectomy (DP) or partial pancreatoduodenectomy (PD)) according to the following three criteria: tumor grade, tumor size and age at operation. All controls were also identified from the prospectively maintained Heidelberg pancreatic database. Functionally active pNEN as well as patients below 18 years of age at the time of operation were excluded from the analysis. Clinical data extraction and follow-up was performed as described above. 

### 2.5. Statistical Analysis

Statistical analyses wer performed using SAS software (release 9.4 SAS Institute, Inc., Cary, NC, USA). The quantitative parameters of age, body mass index (BMI), length of hospital stay, tumor size, length of operation and length of follow-up are presented as median values with an interquartile range (IQR). Because the quantitative parameters are not normally distributed, the non‐parametric Mann−Whitney U test was used to statistically compare cases and controls. The categorical parameters of patient and tumor characteristics, as well as morbidity and perioperative outcomes, are presented as absolute and relative frequencies, and they were analyzed using the Fisher exact test to statistically compare cases and controls.

Overall survival, defined as the time from resection to death from any cause or until the last follow-up, and disease-free survival, defined as the time from resection to the date of detected recurrence or newly diagnosed regional or distant metastases, was analyzed using the Kaplan–Meier method and the log rank test for the comparison of survival curves between cases and controls. Patients alive at the date of last follow-up were censored. Two-sided *p*-values of <0.05 were considered statistically significant. Because of the exploratory character of the analyses performed, all results were interpreted cautiously and the *p*-values were used descriptively. The R optmatch package (version 0.9-15 [49]) was used to perform the case-control match on the propensity score. The propensity scores were calculated using a logistic regression with the matching criteria of grading, tumor size, tumor localization and age as covariables. The pair matching was done using the “match on” function of the optmatch package.

## 3. Results

### 3.1. Patient Characteristics and Matched Cohort

Out of 666 patients resected for a pNEN at our institution between 2001 and 2020, 115 (17.3%) underwent enucleation, 54 of which were functional pNEN. Sixty-one patients were identified as undergoing enucleation of a non-functional pNEN. They were matched with 61 patients with nf-pNEN undergoing formal resection according to age, median tumor size and tumor grading. The clinicopathological characteristics of all patients are presented in Table 1. The median age at the time of operation was 61 years (range 50–68) for enucleation and 62 years (range 52–67) for formal resection. The median tumor size was 1.2 cm for both groups. Fifty-three patients (86.9%) were G1 and eight (13.1%) were G2-differentiated in each group. The male:female ratio was about 1:2 (20 male versus 41 female patients) for enucleation and about 1:1 for formal resection (30 male versus 31 female patients) (*p* = 0.0971). There was also no significant difference in the body mass index of patients (26 versus 27, *p* = 0.1106), nor in the ASA classification (Physical Status Classification System by the American Society of Anaesthesiologists) of patients (ASA I: 3 versus 5 patients, II: 41 patients each; III: 15 versus 12 patients) between the two groups.

Thirty-one patients (50.8%) in the formal resection group underwent PD and thirty (49.2%) underwent DP. The main indications for formal resection were suspicion of malignancy (about 56%) and the technical impossibility of enucleation (26%). There was no significant difference in the amount of laparoscopically or robotically performed procedures (8 (13.1%) in the enucleation group and 11 (18%) in the formal resection group, *p* = 0.6185). The length of operative time was significantly shorter in the enucleation group with 128 min (interquartile range (IQR) 95–170) compared to 263 min (IQR 172–337) in the formal resection group (*p* < 0.0001).

### 3.2. Pathology Data

Analysis of pathology data (see Table 1) showed no significant difference in tumor stage (T1 stage: 44 patients in the enucleation group versus 45 patients in the formal resection group; T2: 15 patients were enucleated and 14 received a formal resection; T3: 2 patients each; *p* = 1.0). There was a significant difference in resection margin status. Microscopically clear margins (R0) upon resection was reached in 45 (73.8%) patients undergoing enucleation compared to 55 (90.2%) undergoing formal resection. R1 status was reached in 9 (14.8%) patients in the enucleation group and 5 (8.2%) in the formal resection group. Also, there was a highly significant difference in the pN status between the two groups (see Table 1). As expected, there was no information on lymph node metastases (pNX) in 42 patients (68.8%) after enucleation due to the lack of lymph node dissection performed on these patients. Selective LND had been performed in some patients undergoing enucleation and thus, 17 patients (27.9%) were pN0 and 2 (3.3%) were pN1, while 57 patients (93.4%) undergoing formal resection were pN0 and 4 (6.6%) were pN1 (*p* < 0.0001). 

### 3.3. Perioperative Outcome

Major postoperative complications (Clavien−Dindo grade III or higher) occurred in 12 patients (19.7%) after enucleation compared to 16 in formally resected patients (26.2%) (Table 2, *p* = 0.5189). Out of these 12 enucleated patients, 11 (18%) had a clinically relevant POPF (8 (16%) with a POPF type B and 3 (2%) with a POPF type C). One patient showed postoperative lower gastrointestinal bleeding requiring endoscopy and blood transfusion. In comparison, a total of 12 (19.7%) patients after formal resection had a POPF (12 (19.7%) had type B and none had type C, *p* = 0.3295). No 30-day and 90-day mortality was observed in the enucleation group and only 1 of the 61 patients died within 30 days after formal resection (*p* = 1.0). This patient, with a known history of coronary heart disease, received a distal pancreatectomy for a G1 NEN. After the development of a POPF type B, this patient died due to cardiac decompensation. The median length of hospital stay after the operation was 8 days (IQR 7–11) compared to 10 days (IQR 8–17), which was not significantly different (*p* = 0.0652). No significant difference was seen in the readmission rate of patients (*p* = 0.6022). Importantly, there was a highly significant difference in the occurrence of postoperative diabetes during long-term follow-up, with only 1 of 61 (1.8%) patients developing diabetes after enucleation, whilst 12 of 61 (20.7%) patients were observed with diabetes type 3c after formal resection (*p* = 0.6022).

### 3.4. Long-Term Outcomes and Survival

**Figure 1:**
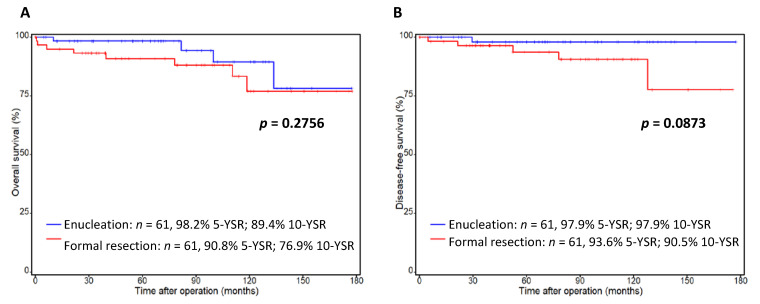
Survival of resected non-functional pNEN—enucleation versus formal resection. Overall survival (**A**) and disease-free (**B**) survival rate of 61 enucleated patients (blue line) and 61 formally resected patients (red line). Exclusion criteria: functional pNEN; Ki-67 > 20%. Patients alive at the point of last follow-up were censored. *p* < 0.05 was determined as the level of significance, OS: overall survival; DFS: disease-free survival. YSR: year-survival rate.

The median follow-up of patients undergoing enucleation was 70 months (IQR 32–111) compared to 85 months (IQR 36–114) after formal resection. Four patients in the enucleation group and eight patients in the formally resected group had died at the last time point of follow-up. The median overall survival was not yet reached in either group (Figure 1A). 5-year overall survival was 98.2% after enucleation and 90.8% after formal resection. After 10 years, OS rate was 89.4% for the enucleation group and 76.9% for the formal resection group. This was not statistically significant (*p* = 0.2756) and the survival curves assimilated after 10 years. For disease-free survival (Figure 1B), the rate was 97.9% 5 years after enucleation and remained at 97.9% after 10 years. For formal resection, the recurrence rate was also low, with a DFS of 93.6% after 5 years and 90.5% after 10 years. Thus, there was a tendency towards slightly better DFS after enucleation. This was not, however, statistically significant (*p* = 0.0873).

## 4. Discussion

This study focused on the perioperative and long-term outcomes of enucleation as a surgical approach for well-differentiated non-functional pNEN.

Analyses so far have focused on the risk of POPF after enucleation compared to the sparing of parenchyma in order to preserve the exocrine and endocrine function of the pancreas. A meta-analysis of 22 studies, including a total of 1148 patients comparing formal resection to enucleation of any pancreatic tumor, showed a higher rate of POPF after enucleation, a significantly lower rate of exocrine pancreatic insufficiency and a shorter median operative time as well as hospital stay. No differences were seen in perioperative mortality or overall morbidity [50]. Similarly, a systematic review of 27 observational studies with a total of 1316 patients comparing enucleation and formal resection of pancreatic tumors were analyzed, showing a reduced rate of postoperative endocrine and exocrine insufficiency, but a significantly higher rate of POPF after enucleation [51]. 

Generally, at our center, pancreatic NEN are considered suitable for enucleation when the tumor size does not exceed 3 cm, the tumor does not obstruct the main duct and the margin is regular. Our group has shown before that enucleation is a safe procedure with a low postoperative morbidity and no mortality in the resection of intraductal papillary mucinous neoplasms [52] and other pancreatic neoplasms [31]. 

For pNEN, specifically, an analysis of 127 enucleated NEN compared to 1009 formally resected pNEN showed a difference in the rate of POPF [53]. Heidsma et al. performed a propensity score matching analysis on 1034 patients undergoing enucleation or formal resection of a pNEN, identified from the databases of two hospitals. In this analysis, comparable long-term outcomes were seen between the two surgical approaches, although enucleation resulted in higher rates of POPF [18]. In this study, however, functional as well as G3-differentiated pNEN were included in the analysis. 

In a propensity score-matched analysis by Weilin et al., the authors analyzed 27 enucleated nf-pNEN patients compared to 94 patients undergoing formal resection [54]. The authors did not observe any 90-day mortality after enucleation. The median follow-up time was 77 months and the 5-year OS and DFS were 100 and 91.4%, which is comparable to the rates observed in our cohort (median follow-up 70 months, 98% 5-year OS and 91% 5-year DFS after enucleation, Figure 1). The median operative time was significantly shorter when patients underwent enucleation (120.33 +/− 106.210 min) compared to when formal resection was performed (224.32 +/− 172.861; *p* ≤ 0.001), which is similar to the results in the present study (128 (95–170) versus 263 (172–337), *p* < 0.0001, Table 1). Further, there was no significant difference in the overall postoperative complication rate, which is in line with the findings of this current study. The authors found a non-significantly higher rate of postoperative diabetes between enucleation and formal resection (27.27% versus 18.52%, respectively). In the present study, this difference was shown to be statistically different in long-term follow-up (2% versus 21%, *p* = 0.0020, Table 2). In the presented analysis, 5-year OS and DFS was excellent for a much larger cohort of enucleated patients (98% OS and DFS). After 10 years, OS was still 89% compared to 77% after formal resection and DFS was still 98% compared to 91% after formal resection (Figure 1). 

Several authors have also proposed active surveillance strategies for small and well-differentiated pNEN [8,13,28,55]. As this study does not include an observational cohort for comparison, no conclusions regarding the natural course of disease can be drawn in this study. However, a recent analysis of 2004 nf-pNEN patients from The National Cancer Database, comparing 1781 resected and 223 non-resected patients, has shown a clearly improved OS after resection of nf-pNEN > 1 cm [29]. This was also seen in a similar analysis including f-pNEN [28]. 

Another treatment option that has increasingly been focused on is radiofrequency ablation (RFA). In 2014, Rossi et al. performed a pilot study that included 10 patients with pNEN < 3 cm and could show that RFA is a feasible option for small pNEN in patients refusing to undergo or are ineligible for surgery [56]. However, 3 of the 10 patients experienced postinterventional pancreatitis and no information on the tumor grades included was available for this study cohort. The median follow-up was 34 months with no evidence of local recurrences in this period of time. Recently, a two center, retrospective study on 27 patients with G1 nf-pNEN showed a rate of peri-interventional pancreatitis of 15% (4/27). Three of those patients had to undergo interventional cystogastrostomy and one patient had to undergo secondary surgery for perigastric fluid collections. The mean follow-up was 15.7 months, and a complete treatment response could be confirmed in 25 of 27 patients [57]. One prospective multicenter study is available, analyzing RFA as a treatment option for several cystic neoplasms of the pancreas. Within the cohort, 12 pNEN patients with tumors < 2 cm were included. The overall postinterventional complication rate was 10%. Follow-up was carried out for one year postintervention. At 1-year of follow-up, there was an 86% treatment success rate for RFA treatment of pNEN [58]. To date, the available data is not sufficient to contextualize RFA as an alternative treatment option. Long-term data are still lacking und an analysis of bigger cohorts is needed. Until then, RFA remains a possibility for a highly selected group of patients that cannot or do not want to undergo surgical treatment.

One important aspect of parenchyma-sparing resection is the fact that this does not preclude the necessity or possibility of a systematic lymphadenectomy. The decision to perform a LND is also still subject to discussion. While some studies have shown a clear association between overall- and disease-free survival with the occurrence of LNM [17,23,24,59,60,61,62], there are other studies disproving this association or only confirming it for a specific subgroup of patients [14,15,62,63].

Regarding the resection margin status, owing to the mere technical nature of enucleating a pancreatic lesion, the resection margin status is expected to be pathologically R1 in a large proportion of patients, since the tumor has been removed along its “capsula”, mostly without additional surrounding healthy/tumor-free pancreatic tissue. Since enucleation is not an oncological resection, this treatment is only used for neoplasms that do not show any features of malignancy, such as metastasis or invasive growth. Additionally, some data thus far have not shown the resection margin status (R0/R1) to be an independent prognostic factor for worse OS or DFS in well-differentiated (enucleated) pNEN [64,65]. Regarding DFS, the lack of role resection margin plays in the presented patient population is most likely an additional selection bias; although, in order to avoid selection bias, matching for age, tumor size and grading had been performed.

The main limitation of this study is its retrospective nature. The data may also be subject to some bias based on the referral and selection of patients for surgery, especially related to the preoperative decision-making for enucleation versus formal resection. One major strength of this paper is the fact that at our hospital, a fluctuating team of 6 experienced pancreatic surgeons perform an annual number of approximately 600 pancreatic resections, thus reducing the likelihood of the non-inferior rate of complications observed in enucleations being related to one particular surgeon’s set of skills. 

To the best of our knowledge, this is the largest single center analysis comparing enucleated well-differentiated nf-pNEN in a nested case-control study with patients undergoing formal resection, analyzing both perioperative surgical and long-term oncological outcomes. 

In order to evaluate patients for the possibility of enucleation, multi-slice CT scan should be performed. MRI/MRCP can be used as an alternative method of cross-sectional imaging. If cross-sectional imaging and radiological expertise is not able to differentiate well-differentiated pNEN from other entities or high-grade pNEN, EUS + FNA/B should be performed. If the tumor is smaller than 3 cm, does not obstruct the main duct, the margin is regular and there are no signs of distant or lymph node metastases, enucleation should be discussed as a treatment option and re-evaluated intraoperatively. Intraoperative assessment of an experienced pancreatic surgeon and the availability of intraoperative ultrasound is a general prerequisite.

From the present results, enucleation of G1- and possibly G2-differentiated nf-pNEN < 3 cm results in excellent long-term outcomes and can be performed with or without LND. 

## 5. Conclusions

This study demonstrates that enucleation can be performed with low morbidity and no mortality in well-differentiated nf-pNEN, with significantly better long-term preservation of the endocrine function of the pancreas compared to formal resection and specifically, no significant differences in the occurrence of postoperative pancreatic fistula and general complication rates. This analysis also shows excellent long-term overall and disease-free survival after enucleation of well-differentiated nf-pNEN. Thus, enucleation of G1- and possibly G2-differentiated nf-pNEN <3 cm is a safe procedure with a non-inferior oncological outcome that should be considered as an alternative surgical approach to formal resection in the absence of lymph node or distant metastases. Enucleation can be performed with LND according to intraoperative finding. More research is needed to determine the role of LND in enucleation. Secondly, preoperative criteria which allow for the selection of enucleation for pNEN patients without compromising the oncological outcome need to be defined. 

## Figures and Tables

**Table 1:** Clinicopathological characteristics of resected pNEN patients (2001–2020).

Parameter	Enucleation *n* = 61	Formal Resection *n* = 61	*p*-Value
Age, Median (IQR)	61 (50–68)	62 (52–67)	0.8840
Median size (IQR) [cm]	1.2 (0.9–2.0)	1.2 (0.8–1.8)	0.8395
Grading (WHO 2017)			1.0
G1	53 (86.9%)	53 (86.9%)	
G2	8 (13.1%)	8 (13.1%)	
Sex [M:F]	20:41	30:31	0.0971
BMI (2 missing values), Median (IQR)	26 (23–29)	27 (25–29)	0.1106
ASA classification (5 missing values)			0.6720
I	3 (5.1%)	5 (8.6%)	
II	41 (69.5%)	41 (70.7%)	
III	15 (25.4%)	12 (20.7%)	
Localization			0.2689
Head	29 (47.5%)	30 (49.2%)	
Body	23 (37.7%)	15 (24.6%)	
Tail	9 (14.8%)	16 (26.2%)	
Laparoscopic/robotic surgery	8 (13.1%)	11 (18.0%)	0.6185
Length of operation [min.], Median (IQR)	128 (95–170)	263 (172–337)	<0.0001 *
pT (TNM 8th)			1.0
T1	44 (72.1%)	45 (73.8%)	
T2	15 (24.6%)	14 (22.9%)	
T3	2 (3.3%)	2 (3.3%)	
R classification			0.0356 *
R0	45 (73.8%)	55 (90.2%)	
R1	9 (14.8%)	5 (8.2%)	
RX	7 (11.5%)	1 (1.6%)	
pN (TNM 8th)			<0.0001 *
N0	17 (27.9%)	57 (93.4%)	
N1	2 (3.3%)	4 (6.6%)	
NX	42 (68.8%)	0 (0.0%)	

* statistically significant; BMI: body mass index; ASA: Physical Status Classification System by the American Society of Anesthesiologists.

**Table 2:** Perioperative outcomes and morbidity.

Parameter	Enucleation *n* = 61	Formal Resection *n* = 61	*p*-Value
30-day mortality	0 (0%)	1 (1.6%)	1.0
POPF			0.3295
BL (biochemical leak)	5 (8.2%)	4 (6.6%)	
B	8 (13.1%)	12 (19.7%)	
C	3 (4.9%)	0 (0.0%)	
POPF B/C	11 (18.0%)	12 (19.7%)	1.0
Clavien−Dindo classification			0.5189
0/I/II	49 (80.3%)	45 (73.8%)	
≥IIIa	12 (19.7%)	16 (26.2%)	
Length of hospital stay (days), Median (IQR)	8 (7–11)	10 (8–17)	0.0652
Postoperative diabetes mellitus (7 missing values)	1 (1.8%)	12 (20.7%)	0.0020 *
Readmission (1 missing value)	7 (11.5%)	9 (15.0%)	0.6022

* statistically significant; POPF: postoperative pancreatic fistula according to ISGPS definition.

## Data Availability

The data presented in this study are available on request from the corresponding author. The data are not publicly available due to data privacy.
